# Case of Death from the Administration of Chloroform

**Published:** 1869-01

**Authors:** E. A. Clark

**Affiliations:** Resident Physician St. Louis City Hospital.


### ARTICLE IX.

### *Case of Death from the Administration of Chloroform.*

By E. A. Clark, M.D., Resident Physician St. Louis City Hospital.

It is my unpleasant duty to report an addition of one more, to the list of fatal results from the administration of chloroform ; and though it is the first serious result that has occurred from the use of this anæsthetic in my hands, in an experience of several years of hospital practice, yet it is none the less to be regretted:

Charles Smith, a native of Ireland, aged 31 years, was admitted to hospital on the 10th of October, affected with hæmorrhoids and prolapsus of the rectum, which had become stangulated external to the sphincter ani. On the following day—Sunday—after a careful examination of the patient, aided by my assistant, Dr. John Bryson, the prolapsed rectum and hæmorrhoidial tumors having become so swollen, tense and painful that he could not tolerate their being handled, I determined to affect their return by placing him under the influence of chloroform. He was apparently in full vigor of health. The heart and lungs were examined and found in a normal condition. The administration of the chloroform was commenced by pouring about a drachm upon a napkin, folded in such manner as to admit the admixture with it of a sufficient quantity of air.

After inhalation had been continued for about two minutes—the pulse and respiration being quite natural, and partial insensibility induced—I commenced to manipulate the tumor, but finding that it still gave him great pain, I directed Dr. Bryson to continue the chloroform, which he did by pouring about one drachm more upon the napkin. Very soon after, the patient began to be affected with the usual spasm, which, however, was not more than ordinarily severe or protracted.

At this moment, while the patient was still struggling, Prof. Robinson, of the Missouri Medical College, entered the room, and assisted Dr. Bryson in administering the chloroform.

In about thirty seconds the muscles began to relax, and the chloroform was discontinued. The pulse was as full and frequent as is usual in a state of complete anæsthesia, and the breathing was quite normal, except slightly stertorous. I observed that his face was more livid than usual, but the favorable condition of his pulse and respiration decided me to proceed with the operation. The patient was turned upon his right side in order to place him in the most favorable position for manipulating the tumor, but in about one minute from the time the chloroform was withdrawn, it was observed that he had ceased breathing, and that the pulse, both at the wrist and carotids, was imperceptible, while the superficial vessels were full and distended, and the face of a dark livid color. He was immediately placed upon his back, the head lowered, and cold water dashed upon the face and chest, with the effect of causing three or four long full inspirations, without, however, affecting the circulation in the least. We then commenced a vigorous artificial respiration, at the same time withdrawing the tongue from the mouth and elevating the epiglottis with the finger.

This was continued without any relaxation for an hour and forty minutes, but without in the least reviving the action of the heart, which, I am confident, never beat again from the moment that natural respiration ceased; he was dead from that instant.

The *post-mortem*, made eighteen hours after death, revealed a considerable serus exudation beneath the arachnoid, which was doubtless the result of the venous congestion of the brain which was found to to exist; the ventricles were empty, containing scarcely a drop of blood, while all the valves, as well as the walls of the heart, were in a perfect healthy condition. The lungs were likewise healthy, presenting, however, some hypostatic congestion on their posterior surface.

From the above detail of facts, the immediate cause of death in this case would seem indeed obscure, though probably not more so than in others not attributable to organic lesions. From, however, the empty condition of the heart, it would be most reasonable to suppose that death was the result of sudden spasmodic contraction of the heart, which continued until life was extinct, this probably being the effect of the anæsthetic upon the ganglionic nervous system. At all events we can hardly suppose it to have been the result of syncope, as is generally thought to be the rationale of most cases of death occurring from the administration of chloroform. This seems to be apparent in the present case, from the congested condition of the blood vessels of the brain, as well as from the fact that we failed to resusicate him by lowering the head beneath the level of the body, a method so generally successful, where a condition of syncope is supposed to exist, in cases threatening death.—*Humboldt Med. Archives.*

